# Frequency of fruit consumption and savoury snacking predict psychological health; selective mediation via cognitive failures

**DOI:** 10.1017/S0007114522001660

**Published:** 2023-02-28

**Authors:** Nicola-Jayne Tuck, Claire V. Farrow, Jason Michael Thomas

**Affiliations:** School of Psychology, Aston University, Birmingham B4 7ET, UK

**Keywords:** Fruit, Vegetables, Snacking, Cognition, Psychological health, Mental health, Wellbeing

## Abstract

While there is growing interest in the link between diet and psychological health, there is a surprising lack of studies investigating the precise associations between nutrient-rich foods (such as fruit and vegetables) *v*. nutrient-poor foods (such as energy-dense savoury and sweet snacks), and psychological health. Similarly, the psychological processes underpinning the relationship between dietary intake and psychological health remain unclear. Hence, the present study aimed to explore the relationship between dietary consumption and psychological health, with cognitive processes as a theoretical mediator. This cross-sectional online study included 428 healthy adults (53 % female; mean age = 39·7 years, sd = 13·0), with participants completing a range of validated questionnaires measuring dietary habits and psychological health. Stepwise multiple regression revealed that more frequent consumption of fruit was associated with reduced symptoms of depression (*β* = –0·109, *P* = 0·025) and greater positive psychological wellbeing (*β* = 0·187, *P* < 0·001). Conversely, more frequent savoury snacking was associated with increased anxiety (*β =* 0·127, *P* = 0·005). Further, mediation analyses revealed that more frequent consumption of savoury snacks was associated with increased symptoms of depression, stress, anxiety and reduced psychological wellbeing, via an increase in cognitive failures (*ps* < 0·001). These results provide new insights on the independent associations between certain types of food and psychological health, and the psychological mechanisms that may mediate these. Further work is now required to establish causality and determine whether these may represent modifiable dietary targets that can directly (and indirectly) influence our psychological health.

Psychological health includes the absence of mental health complaints and the experience of positive psychological wellbeing^(1)^. Given the health, social and economic burden of impaired psychological health, there are calls for new preventative public health approaches^(2)^, with recent research suggesting that dietary intake is a potential target for improving psychological health^(3)^.

Indeed, the consumption of nutrient-rich (unprocessed) foods, such as fruit and vegetables, have been associated with fewer psychological health issues^(4,5)^ and reduced cognitive impairment^(6)^. Fruit and vegetable intake (FVI) are also associated with a reduced risk of symptoms of depression, stress and anxiety^(7,8)^, and there is accumulating evidence that FVI is positively related to psychological wellbeing^(9,10)^. Although studies have begun to explore fruit and vegetable consumption as separate predictors of psychological health^(11,12)^, few have evaluated the impact of frequency. However, a recent study reported that the frequency with which fruits and vegetables are consumed may be more important than quantity of consumption, suggesting a more nuanced approach to consumption may be required^(10)^.

Conversely, habitual consumption of nutrient-poor (processed) foods, such as sweet and savoury snacks, is associated with increased risk of depression, anxiety, stress^(13)^ and lower psychological wellbeing^(14)^. A prospective study reported that *trans*-unsaturated fatty acid in processed food, such as snack foods (crisps, cookies, cakes, etc.), was associated with a higher depression risk at follow-up of 8–10 years later risk^(15)^, illustrating the potential long-term effects of diet on psychological health. Further, evidence shows that a decrease in frequency of fruit intake and an increase in frequency of snack food intake independently coincided with increased perceived stress^(16)^. Thus, the frequency of nutrient-poor food consumption may also distinctively contribute to psychological health.

While little is known about the psychological mechanisms by which diet may affect psychological health, the role of cognitive processes (e.g. memory and executive function) has been implicated^(17,18)^. Dietary intake high in energy content or low in micronutrients, antioxidants or fibre consumption may reduce optimal brain function, such as neurotransmitter regulation and inflammatory pathways, leading to poorer psychological health^(19)^. FVI have been shown to enhance cognition^(20)^, whereas saturated fat and sugar intake decrease cognitive performance^(21)^. Specifically, diet-induced changes to memory and inhibitory control have been consistently observed^(22,23)^. Frequent cognitive failures (memory errors) have been associated with increased perceived stress and sadness^(24,25)^. Additionally, reduced inhibitory control is a risk factor for depression^(26,27)^. Even subtle cognitive deficits are significantly associated with reduced psychological wellbeing^(28)^; therefore, dietary intake may impact cognition and in turn psychological health.

Research has begun to identify relationships between the consumption of nutrient-rich or nutrient-poor foods and psychological health. However, it is important to evaluate the independent associations of the frequency and quantity of FVI, frequent snacking on energy-dense foods, and the role of cognitive processes as a potential mediator. This study aimed to assess the direct and indirect relationship between dietary intake and depression, anxiety, stress and wellbeing. It was predicted that greater nutrient-rich FVI (as separate frequency and portions variables) would be associated with increased psychological health, whereas greater nutrient-poor sweet and savoury snacking would be associated with decreased psychological health. It was also predicted that cognitive failures and inhibitory control would mediate the relationship between dietary intake and psychological health, whereby FVI would negatively predict cognitive failures and positively predict inhibitory control scores and thus increase psychological health, whereas, sweet and savoury snacking would positively predict cognitive failures, negatively predict inhibitory control, and thus reduce psychological health.

## Method

### Participants

Participants were recruited using the recruitment platform Toluna^(29)^ to gain access to a nationally representative sample of the UK. Toluna is an online community website which invites members to complete paid surveys. Participants were compensated with 3000 Toluna points (redeemable towards retail vouchers) after completing the survey. A total of 977 participants provided informed consent to take part, with 442 participants completing this online cross-sectional study. Of these, fourteen were excluded from analyses due to having a go reaction time percentage accuracy lower than 60 % on the inhibitory control task (see below for more information on this task), thus the total n for analysis = 428 (53 % were female). Participants completed an initial screener questionnaire to ensure they met the inclusion criteria which included being aged 18–60 years of age, not colour blind (due to the demands of the inhibitory control task) and having English as their first language. Participants who rated their general health as poor over the last 12 months, who currently have or had diabetes, or an eating disorder and/or medically diagnosed food allergy, high blood pressure, a heart attack, or were experiencing medical illness were not eligible to participate (509 participants did not meet the screening criteria and 26 eligible participants left the survey incomplete). The study was approved by the College of Health & Life Sciences Ethics committee at Aston University.

### Sample size

Using G × Power 3.1.9.2, with *α* set at 0·05, modelling a small effect size (*f* = 0·02), and power at 80 %, the minimum required sample size was 395 participants^(30)^. However, to account for participants who might not complete the study in full, or whose data may need to be excluded from analysis (e.g. due to the threshold for the inhibitory control task), we aimed to enrol a minimum of 450 participants onto the study.

### Procedure

The Gorilla Experiment platform was used to create and host the cross-sectional study. The survey and cognitive task were completed by participants online using a computer; phones or tablets were not permitted because the cognitive task required the use of a keyboard. Participants who expressed a willingness to take part provided informed consent and were screened for the inclusion criteria. Eligible participants then progressed through the series of questionnaires measuring demographic information, dietary intake, lifestyle behaviours, psychological health, cognition, mood and appetite, followed by the stop-signal task (SST), and were finally debriefed.

### Measures

#### Lifestyle behaviours, health and demographic information

During an initial screener questionnaire, general health was assessed using a single item with a five-point Likert scale (ranging from poor to excellent) asking participants to rate: ‘Over the last 12 months, would you say that on the whole, your health has been…’. Participants were also asked if they previously or currently had diabetes, an eating disorder, a medically diagnosed food allergy, high blood pressure, a heart attack or any other medical illness in order to recruit healthy adults. Participants were asked their age and if they were colour blind (due to the demands of the cognitive task) during the initial screener questionnaire to meet the inclusion criteria. Sex, ethnic group, years and level of education and household income data were also collected to characterise the sample.

Within the short-form FFQ (see below), there were items that assessed average weekly alcohol intake, exercise and smoking status. Exercise (total minutes last week) was used in the present study as a control variable for the mediation models, given the evidence that physical activity has beneficial effects on cognitive function^(31,32)^ and psychological health^(33)^. Additionally, the twenty-one-item, four-point Likert Three-Factor Eating Questionnaire (TFEQ)^(34)^ was used to collect data on eating style in order to calculate an uncontrolled eating, cognitive restraint and emotional eating average score for the sample. Participants were also asked if they were vegetarian or vegan. To calculate BMI, participants were asked to report their height and weight in either metric or imperial units.

#### Dietary intake

The short-form FFQ was used to examine the consumption of foods in a ‘typical’ week, over the past month by measuring the frequency of food group consumption using an eight-point Likert scale (rarely or never to 5 + a day)^(35)^. The food groups investigated in this study were as follows: (1) fruit consumption, which included fresh or tinned; (2) vegetable consumption, which included fresh, tinned or frozen, but not potatoes; (3) sweet snacking, which included biscuits, cakes, chocolate and sweets, and; (4) savoury snacking, which included crisps or savoury snacks. In addition to these measures of frequency of consumption, the short-form FFQ included two additional single items to collect data on average fruit and vegetable consumption per day in portions. One portion was quantified as approximately 80 g in weight and examples were provided for each item, such as ‘a handful of grapes’. The measure did not include similar items for sweet or savoury snacking; however, this questionnaire is valid and reliable for assessing diet in the UK population, hence, was used here^(36)^. Validation of this measure has shown that participant short-form FFQ responses for single food items are independently predictive of a participant’s diet quality score (as measured by a 217-item FFQ used in the UK Women’s Cohort Study)^(36)^. This highlights that individual food groups are significant for dietary assessment.

#### Psychological health

Symptoms of depression and anxiety during the past week were measured using the fourteen-item, four-point Likert, Hospital Anxiety and Depression Scale^(37)^. The Hospital Anxiety and Depression Scale is recommended for use in the general population as it was designed as a screening tool to identify possible and probable cases of anxiety and depression in outpatients^(38)^. Stress was measured using the fourteen-item, five-point Likert, Perceived Stress Scale^(39)^. The items examine levels of stress experienced over the last month. The Perceived Stress Scale was designed for use within community samples^(39)^. Visual analogue scales were used to assess current mood and appetite ratings^(40)^, and these were included immediately before the cognitive task. Participants indicated on a 100-mm horizontal line the point that represented their current experience of the following (where 0 = not at all, and 100 = very much): sad, happy, anxious, alert, drowsy, withdrawn and hungry. Positive psychological wellbeing was measured using the fourteen-item, five-point Warwick–Edinburgh Mental Wellbeing Scale, which is validated for use in the general adult population^(41)^. The Warwick–Edinburgh Mental Wellbeing Scale evaluates subjective wellbeing and psychological functioning over the last 2 weeks^(42)^.

#### Cognition

The twenty-five-item self-report Cognitive Failures Questionnaire (CFQ) measured attentional, memory, perceptual and action-related mental lapses in everyday tasks over the past 6 months^(43)^. One item was adapted: ‘Do you leave important letters unanswered for days?’ whereby ‘letters’ was changed to ‘emails’. The CFQ was used to assess the frequency of global cognitive dysfunction. Scores can range from 0 to 100, whereby higher scores indicate more subjectively experienced cognitive failures. The CFQ has high internal validity (α = 0·91) and test–retest reliability^(44)^. The CFQ has been correlated with the Everyday Memory Questionnaire (*r* = –0·64) which assesses memory errors^(45)^.

The SST is a behavioural measure of inhibitory control which was used to provide a Stop-signal Reaction Time (SsRT); this measure represents a participant’s efficiency to inhibit an already initiated response^(46)^. Higher SsRT indicate poorer inhibitory control, and the SST has been shown to be one of the most sensitive and reliable measures of executive control^(47)^. This task-based measure of inhibitory control may provide insight into the specific neural and behavioural impairments of cognition associated with both eating and the development or maintenance of poor psychological health^(48–50)^. An online SST was created using The Gorilla Experiment Builder^(51)^ based on the stop-signal paradigm^(52)^. The SST included a practice task with sixteen trials whereby participants were required to reach an accuracy threshold of 50 % to proceed to the main task. The main task comprised six blocks of sixteen trials (ninety-six trials in total, 75 % go trials, 25 % stop-signal trials). Each trial started with the presentation of a fixation cross which was replaced with an arrow pointing in the left or right direction inside a white circle (the inter-stimulus interval was 500 ms). Participants indicated the direction of the arrow using the keyboard responses: ‘b’ for left and ‘n’ for right (the maximum response time was 1500 ms before the trial moved along). On stop-signal trials, the white circle surrounding the directional arrow changed to red which occurred after a variable delay ranging from 250 to 400 ms, with 50 ms incremental increases in difficulty, and participants were required to withhold a response on these trials.

### Statistical analyses

**Fig. 1.**
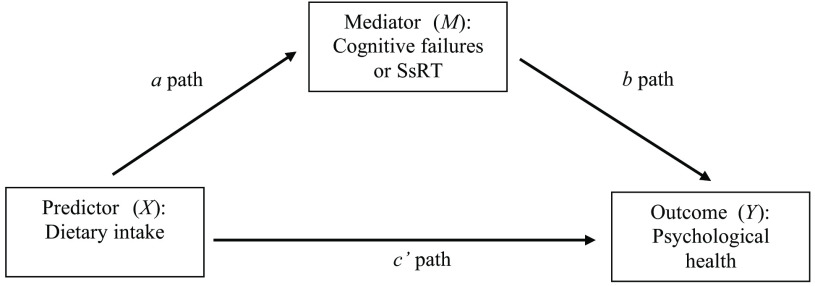
Mediation paths. All models controlled for general health rating and exercise. SsRT, Stop-signal Reaction Time.

Descriptive data were calculated as means with standard deviations, or as frequencies (%) for categorical variables. For the frequency of dietary intake, the median and range are presented. For processing the cognitive task raw data, a published protocol was applied to estimate SsRT and the average stop-signal delay^(53)^. Simple linear and stepwise multiple regression analyses were conducted to explore the relationship between dietary intake and psychological health (before and after including the covariates: age, sex, BMI, exercise, general health rating, smoking status and alcohol intake). The selection of these covariates was informed by previous literature^(7,10,11)^. The stepwise analysis put predictors into competition with each other by applying the ≤ 0·050 to enter and ≥ 0·1 to remove criteria. Separate models were conducted for dietary intake (food frequency) for fruit, vegetable, sweet and savoury snack consumption as predictors. FVI (separately) quantified as portions consumed were also explored as separate predictors. The outcome variables included four different measures of psychological health: symptoms of depression, anxiety, stress and positive psychological wellbeing scores. Multiple mediation analyses were performed using PROCESS, version 3.5^(54)^ in SPSS version 26 using bootstrapping over 5000 samples. The mediators included in the models were either cognitive failures score or SsRT (the index of inhibitory control). All mediation models included covariates that were consistently, significantly associated with the psychological health outcomes at the *P* < 0·01 level, which were general health rating and exercise (see Fig. 1 for the mediation paths).

## Results

### Participants characteristics

Descriptive statistics regarding demographic and lifestyle behaviour information for the healthy adult sample are presented in Table 1. On average, the participants were middle aged, 52·8 % of the sample were female and 89·7 % identified as White for ethnicity. The mean BMI for the sample was 26·0 (sd = 5·6) and 53·5 % were normal weight. On average, participants exercised for 2·5 h per week and most of the sample rated their general health as good to excellent (86 %). Further, 63·1 % were not smokers and 79·2 % consume less than 14 units of alcohol per week or rarely/never drink. Finally, education and household income information were provided by some of the sample (*n* 207), and of these, the majority achieved university-level education (66 %), and their household income was between £20 000 and £49 999 (73 %).

**Table 1.** Sample demographic information and lifestyle behaviour (numbers and percentages; mean values and standard deviation, *n* 428)

Characteristics	*n*	Mean	sd	Percentage/range
Mean age in years		39·7	13·0	18–60
Sex				
Female	226			52·8
Male	200			46·7
Other	2			0·5
Ethnicity/race				
White	384			89·7
Asian	8			1·9
Black	13			3·0
Chinese	7			1·6
Mixed	10			2·3
Other	5			1·3
Prefer not to say	1			0·2
Mean BMI		26·0	5·6	14–50
Underweight	12			2·8
Normal weight	229			53·5
Overweight	103			24·1
Obese	78			18·2
Prefer not to say	6			1·4
Mean exercise (total minutes)		152·9	151·1	0–840
General health rating				
Excellent	62			14·5
Very good	167			39·0
Good	140			32·7
Fair	59			13·8
Smoking				
Never smoked > 100 cigarettes	270			63·1
Current smoker	77			18·0
Ex-smoker	81			18·9
Alcohol intake (units per week)				
Rarely or never drink	170			39·7
< 14 units	169			39·5
14–21 units	56			13·1
> 21 units	33			7·7
Eating style (TFEQ)				
Uncontrolled eating score		2·4	0·6	1–3·8
Cognitive restraint score		2·6	0·5	1–3·8
Emotional eating score		2·6	0·9	1–4·0

TFEQ, Three-Factor Eating Questionnaire.

BMI *n* 422 because six participants selected ‘prefer not to say’.

NHS classifications for BMI ranges were used; underweight < 18·5, normal weight 18·5–24·9, overweight 25–29·9, obese ≥ 30; eating style (TFEQ) average scores for each dimension (uncontrolled eating, cognitive restraint and emotional eating) indicate a non-disordered sample.

### Dietary intake

The frequency of dietary intake showed that on average, both sweet and savoury snacks were each consumed 2–3 times a week, while both fruit and vegetables were each consumed 4–6 times a week. The range of responses was from rarely or never to 5+ times a day; overall participants were not consuming fruit and vegetables frequently enough to reach recommended intake. On average, participants consumed 1·9 portions of fruit per day (sd = 1·5) and 2·3 portions of vegetables per day (sd = 1·5); thus, combined consumption was short of the recommended daily intake. FVI ranged from 0 to 10 portions per day and 10% of the sample identified as vegetarian or vegan.

### Cognition

For the CFQ, the average frequency, variance and range of cognitive failures scores were as follows: mean = 34·8, sd = 16·2 and range = 0–97. For the SST, fifty-three participants (12 % of the sample) were required to complete the practice trials a second time to meet the accuracy threshold of 50 % and proceed to the test phase. However, descriptive results from the test phase showed that on average, the percentage accuracy on stop-signal and go trials was high (stop-signal trials, mean = 81 %, sd = 23 %; go trials, mean = 93 %, sd = 9 %). Finally, calculation of the SsRT revealed a mean of 151·4 ms (sd = 276·1 ms).

### Psychological health

Descriptive statistics for mood ratings and psychological health are presented in Table 2. Visual analogue scale scores show that on average participants were happy, alert, low in sadness and anxiousness and not highly drowsy or withdrawn. Overall, mean depression and anxiety scores were considered non-clinical and stress levels were moderate^(38,55)^. The mean psychological wellbeing score (46·0) is similar to other UK general population groups^(56)^. The Warwick–Edinburgh Mental Wellbeing Scale does not a have a ‘cut-off’ level to indicate those who have ‘good’ and those who have ‘poor’ psychological wellbeing, but the minimum score is 14 and the maximum is 70^(42)^.

**Table 2.** Descriptive results for mood ratings and psychological health (Mean values and standard deviation, *n* 428)

Outcome	Mean	sd	Range
Sad (VAS)	26·2	25·2	0–100
Happy (VAS)	61·7	23·2	0–100
Anxious (VAS)	28·0	26·6	0–100
Alert (VAS)	68·3	22·1	0–100
Drowsy (VAS)	36·0	26·8	0–100
Withdrawn (VAS)	33·2	29·1	0–100
Hungry (VAS)	31·2	28·2	0–100
Depression score (HADS)	6·8	3·6	0–18
Anxiety score (HADS)	7·3	4·5	0–21
Stress score (PSS)	16·9	7·8	0–40
Psychological wellbeing (WEMWBS)	46·0	10·3	14–70

VAS, Visual analogue scale; HADS, Hospital Anxiety and Depression Scale; PSS, Perceived Stress Scale; WEMWBS, Warwick–Edinburgh Mental Wellbeing Scale.

### Regression results

#### Simple linear regression

Prior to the inclusion of covariates, simple linear regression was conducted to analyse sweet and savoury snacking, fruit, and vegetables (frequency or portions) consumed as predictors of either depression, anxiety, stress or wellbeing scores. Frequency of fruit consumption and portions of vegetables consumed negatively predicted depression scores (*β =* –0·140, *P* < 0·05, *R^2^ =* 0·020; *β =* –0·108, *P* < 0·05, *R² =* 0·012, respectively). The results also revealed that frequency and portions of fruit consumption positively predicted psychological wellbeing scores (*β =* 0·192, *P* < 0·001, *R² =* 0·037; *β =* 0·120, *P* < 0·05, *R² =* 0·014, respectively). Furthermore, sweet and savoury snacking positively predicted anxiety scores (*β =* 0·098, *P* < 0·05, *R² =* 0·010; *β =* 0·186, *P* < 0·001, *R² =* 0·035, respectively), while only savoury snacking positively predicted stress (*β =* 0·136, *P* < 0·05, *R² =* 0·019). Frequency of vegetable consumption did not predict psychological health (all *p*s > 0·05).

#### Stepwise regression

To evaluate the relative contribution of dietary intake (sweet and savoury snacking, frequency and portions of fruit and vegetable consumption) as predictors of psychological health, compared with other known predictors, the following covariates were included in a stepwise multiple regression: age, sex, BMI, exercise, general health rating, smoking status and alcohol intake. The significant stepwise regression models’ β values, *P* values, change in *R²*, and the adjusted *R²* for each step of the analysis are presented in Table 3. Firstly, for depression scores, the significant stepwise model selected exercise for entry first (*β =* –0·112*, P* = 0·021, *R² =* 0·027), then age was added (*β =* –0·180, *P* < 0·001, *R² =* 0·047), followed by general health rating (*β =* –0·128, *P* = 0·008, *R² =* 0·068), smoking status (*β =* –0·112, *P* = 0·019, *R² =* 0·084) and finally fruit frequency (*β =* –0·109, *P* = 0·025, *R² =* 0·095). Overall, the model predicted 8·4 % of the variance in depression scores (*F* (5, 416) = 8·735, *P* < 0·001, adjusted *R²* total = 0·084). Secondly, for wellbeing scores, the significant stepwise model selected general health rating for entry first (*β =* 0·255, *P* < 0·001, *R² =* 0·064), then fruit frequency (*β =* 0·187, *P* < 0·001, *R² =* 0·096) and finally age (*β =* 0·140, *P* = 0·003, *R² =* 0·116). Overall, the model predicted 10·9 % of the variance in wellbeing scores (*F* (3, 418) = 18·199, *P* < 0·001, adjusted *R²* total = 0·109). Finally, for anxiety scores, the significant stepwise model selected age for entry first (*β =* –0·311, *P* < 0·001, *R² =* 0·105), then general health rating was added (*β =* –0·136, *P* = 0·003, *R² =* 0·130), followed by smoking status (*β =* –0·139, *P* = 0·002, *R² =* 0·148), savoury snacking (*β =* 0·127, *P* = 0·005, *R² =* 0·163) and finally sex (*β =* 0·101, *P* = 0·029, *R² =* 0·172). Overall, the model predicted 16·2 % of the variance in anxiety scores (*F* (5, 416) = 17·296, *P* < 0·001, adjusted *R²* total = 0·162).

**Table 3.** The significant stepwise regression models for the relationship between fruit frequency, savoury snacking and psychological health, including covariates (*n* 428)

Depression	Exercise	Age	General health rating	Smoking status	Fruit frequency
Unstandardised β	–0·003	–0·050	–0·507	–0·519	–0·188
Standardised β	–0·112	–0·180	–0·128	–0·112	–0·109
*P*	0·021	< 0·001	0·008	0·019	0·025
	Step 1	Step 2	Step 3	Step 4	Step 5
R, *R²*	0·164, 0·027	0·216, 0·047	0·262, 0·068	0·290, 0·084	0·308, 0·095
*R²* change	0·027	0·020	0·022	0·016	0·011
Adjusted *R²*	0·025	0·042	0·062	0·075	0·084
Wellbeing	General health rating	Fruit frequency	Age		
Unstandardised β	2·888	0·916	0·110		
Standardised β	0·255	0·187	0·140		
*P*	< 0·001	< 0·001	0·003		
	Step 1	Step 2	Step 3		
R, *R²*	0·254, 0·064	0·310, 0·096	0·340, 0·116		
*R²* change	0·064	0·032	0·019		
Adjusted *R²*	0·062	0·092	0·109		
Anxiety	Age	General health rating	Smoking status	Savoury snacking	Sex
Unstandardised β	–0·108	–0·677	–0·805	0·362	0·898
Standardised β	–0·311	–0·136	–0·139	0·127	0·101
*P*	< 0·001	0·003	0·002	0·005	0·029
	Step 1	Step 2	Step 3	Step 4	Step 5
R, *R²*	0·324, 0·105	0·361, 0·130	0·385, 0·148	0·403, 0·163	0·415, 0·172
*R²* change	0·105	0·025	0·018	0·014	0·010
Adjusted *R²*	0·103	0·126	0·142	0·155	0·162

Note: Depression: Step 1 exercise; Step 2 age was added; Step 3 general health rating added; Step 4 smoking status added; Step 5 fruit frequency added. Wellbeing: Step 1 general health rating; Step 2 fruit frequency was added; Step 3 age added. Anxiety: Step 1 age; Step 2 general health rating was added; Step 3 smoking status added; Step 4 savoury snacking added; Step 5 sex added.

Focussing on dietary intake within these models, fruit frequency was a significant negative predictor of depression scores (*β =* –0·109, *P* = 0·025) and independently contributes 1·1 % to the variance explained (*R²* change value at step 5 = 0·011), with an adjusted, standardised β estimate of –0·099 (after adjusting for exercise, age, general health rating and smoking status retained in the significant stepwise model). Fruit frequency was also a significant positive predictor of positive psychological wellbeing scores (*β* = 0·187, *P* < 0·001) and independently contributes 3·2 % to the variance explained (*R²* change value at step 2 = 0·032), with an adjusted, standardised β estimate of 0·180 (after adjusting for general health rating and age retained in the significant stepwise model). The unstandardised β values (presented in Table 3) show that for every 1 unit increase in the frequency of fruit consumption (e.g. from 4–6 times a week to 1–2 times a day), depression scores decrease by 0·188, while positive wellbeing scores increase by 0·916. Furthermore, savoury snacking was a significant positive predictor of anxiety scores (*β* = 0·127, *P* = 0·005) and independently contributes 1·4 % to the variance explained (*R²* change value at step 4 = 0·014), with an adjusted, standardised β estimate of 0·131 (after adjusting for age, general health rating, smoking status and sex retained in the significant stepwise model). For every 1 unit increase in the frequency of savoury snacking (e.g. from 2–3 times a week to 4–6 times a week), anxiety scores increase by 0·362 (unstandardised β value). After including the covariates in the stepwise regression, all other models for snacking, vegetable and fruit portions were no longer significant predictors of psychological health (all *p*s > 0·05).

### Mediation results

Mediation analyses were applied to follow-up on the significant regression analyses presented above. Cognitive failures mediated the relationship between savoury snacking and psychological health whilst controlling for general health rating and exercise. Specifically, savoury snacking significantly positively predicted cognitive failures (*a* pathway; *B* = 1·93, *P* < 0·001). As cognitive failures increased, so did depression (*b* pathway; *B* = 0·10, *P* < 0·001), stress (*b* pathway; *B* = 0·23, *P* < 0·001) and anxiety scores (*b* pathway; *B* = 0·16, *P* < 0·001), whereas wellbeing scores decreased (*b* pathway; *B* = –0·23, *P* < 0·001). Savoury snacking was not a direct significant predictor of psychological health when cognitive failures were held constant (for all *c’* pathways, *P* > 0*·*05); however, savoury snacking was a significant predictor of stress (*P* < 0·05) and anxiety (*P* < 0·001) when the indirect and direct pathways were combined (*c* total pathway). Further, examining the indirect effect (*ab* pathway) indicated that mediation had occurred for: depression (*ab* = 0·188, 95 % CI [0·082, 0·309]); anxiety (*ab* = 0·295, 95 % CI [0·134, 0·472]); stress (*ab* = 0·436, 95 % CI [0·196, 0·691]) and wellbeing (*ab* = –0·433, 95 % CI [–0·721, –0·188]). No further significant mediation was identified, either for the other predictors (dietary predictors) or for the other theoretical mediator (inhibitory control). The models did reveal that higher SsRT (indicative of poorer efficiency inhibiting a response) was associated with a significant increase in depression (*P* < 0·001) and stress (*P* < 0·05) scores.

## Discussion

This study assessed the direct relationship between dietary intake and psychological health, while extending existing literature by evaluating both frequency and portions of fruit and vegetable consumption, separately. It also explored a novel mediation model to evaluate the indirect relationship between dietary intake and psychological health via cognitive processes. Results showed that after including a range of covariates, fruit frequency negatively predicted depression scores and positively predicted psychological wellbeing scores. By contrast, savoury snacking positively predicted anxiety scores. Although, cognitive failures did not mediate the relationship between either FVI or sweet snacking and psychological health, mediation was observed for savoury snacking, whereby more frequent consumption of savoury snacks was associated with greater cognitive failures, and in turn, reduced psychological health, including increased symptoms of depression, stress and anxiety, and lower positive psychological wellbeing. Inhibitory control did not mediate any of the relationships between dietary intake and psychological health. The results emphasise that frequency of fruit consumption and savoury snacking could be potential targets for improving psychological health at the individual level, which in turn could have larger gains (e.g. health, social and economic) at a population level.

The positive relationship between FVI and psychological health has been consistently reported in the literature^(57,58)^; however, few studies have analysed FVI separately^(59,60)^, and even fewer have compared frequency and portions (quantity)^(10)^. The current results provide further nuance by revealing that the frequency with which fruit is consumed, but not the portions consumed during a typical week, negatively predicted depression and positively predicted psychological wellbeing, after including covariates. This suggests that how often we consume fruit may be more important than the total amount we consume. Indeed, poor mental health has been associated with less frequent intake of fresh fruits among women and men^(61)^. Additionally, frequent consumption of fruit snacks over a 2-week period has been shown to improve positive psychological wellbeing^(62)^. Hence, the present findings support the notion that frequently consuming nutrient-rich fruits may be more important than the quantity of consumption for psychological health^(10)^; however, experimental studies, varying frequency and portions of fruit consumed, are required to test this directly.

Although fruit frequency predicted psychological health, it is notable that neither the frequency of consumption, or the portions of vegetables consumed, were significant predictors when including age, sex, BMI, exercise, general health rating, smoking status and alcohol intake as covariates. This highlights not only the importance of covariate analysis but also the significance of assessing FVI individually. Furthermore, FVI are often consumed in different environmental contexts as vegetables are typically found to be consumed with family members at home during meal times, while fruits are typically consumed as snacks outside the home and throughout the day^(63)^. Although the null association for vegetables and psychological health was not predicted, it is not necessarily surprising. Recent research suggests that the relationship between FVI and psychological health is stronger for raw fruit and vegetables compared with cooked or canned^(64)^, and that frequent intake of fruit may involve greater consumption in raw form (for instance, snacking on whole fruits), which may maximise the absorption of nutrients with antioxidant properties, thus having a more potent influence on psychological health^(65)^.

Further, precise quantities (daily portions) of vegetable intake may be required to observe an influence on psychological health. Previous research demonstrated that an association between vegetable portions consumed and reduced symptoms of depression only occurred at higher levels of intake every day (≥ 5·0 portions/d)^(11)^. Thus, the current sample’s low average vegetable consumption (2·3 portions/day), offers an alternative explanation of the null association, here. Further, recent findings suggest that certain types of fruits and vegetables may be more effective in reducing symptoms of depression. For instance, intakes of tomatoes, dark-green vegetables, berries and fruits were more strongly negatively related to symptoms of depression, than other vegetables and dried fruits^(66)^. Therefore, higher quantities of certain categories of vegetables may need to be consumed in order to observe benefits to psychological health.

In contrast to the patterns described above, savoury snacking positively predicted anxiety scores (but neither FVI or sweet snacking predicted anxiety scores). Previous research has explored snacking more broadly by combining sweet and savoury intake^(13)^; thus, the current results shed light on a specific link between savoury snacking and anxiety, after including covariates. These results support previous work demonstrating that poorer dietary intake including higher salty snack and fast-food consumption is associated with greater anxiety^(67)^. Of course, it is necessary to point out that the direction of this relationship is uncertain, as a strategy to cope with anxiety often involves increased consumption of nutrient-poor foods^(68)^. However, it may be the case that savoury snacking is more problematic for psychological health than sweet snacking because the current results suggest that a robust direct and indirect relationship exists. Hence, similar to fruit and vegetables, assessing the individual contributions of sweet and savoury snacking to our psychological health appears to be warranted.

Following on from this finding, we also revealed that more frequent savoury snacking was associated with an increase in cognitive failures, which were in turn associated with increased symptoms of depression, stress and anxiety, but also a decrease in wellbeing. This mediation supports previous findings that snacking on nutrient-poor processed foods, such as crisps, is associated with increased cognitive and psychological problems^(13,14)^ and builds on findings that reported a positive relationship between frequent unhealthy snacking and higher cognitive failures and stress^(69)^. Research shows that subjective memory concern during daily activities is a significant predictor of psychological wellbeing, anxiety and symptoms of depression^(28)^. It is possible that savoury snacking may have a negative effect on psychological health, via a decrease in cognition related to general memory lapses due to saturated fat content^(22,70)^. There is an abundance of evidence from animal models showing that high saturated fat diets specifically reduce memory function^(71)^ which speaks to the causal link between diet and cognition. Hence, the memory errors reflected in the cognitive failures measure may be the cognitive mechanisms by which processed food intake indirectly reduces psychological health. Indeed, that inhibitory control did not mediate this effect may suggest that specific cognitive processes are involved, relating to memory. Thus, future work should focus on delineating the precise cognitive mechanisms involved, and establishing causality, using an experimental approach.

Strengths of this study included that it was a large national sample of the UK population, including a wide participant age range with each sex well-represented. Also, instead of a general measure of psychological health, multiple aspects were measured as discrete outcomes (depression, anxiety, stress and positive psychological wellbeing), and similarly, mutiple aspects of dietary intake were assessed (fruit and vegetables separately; frequency and portions, etc.) providing greater precision. Nevertheless, there are limitations to be considered. This study provides a credible model by which we can infer causal relationships, but due to the cross-sectional design, further prospective and experimental work is required to test causality and temporality robustly. Furthermore, the food frequency measure did not identify the number of snacks consumed; therefore, the portions of sweet and savoury snacks consumed were not evaluated. Relatedly, this measure could also benefit from validation with biomarker levels to establish the level of agreement. Finally, the testing environment could not be precisely controlled, as this was an online study; thus, the results must be considered with that caveat in mind. However, the results suggest that psychological health can be directly and indirectly influenced by specific nutrient-rich (fruit) and nutrient-poor foods (savoury snacks) which contribute to an evidence base for developing effective preventive strategies in public health. Promoting dietary habits that lead to better psychological health could improve individual wellbeing, alleviate strain on healthcare systems and reduce the economic cost associated with both poor psychological health and cognitive failure^(68)^.

In conclusion, this study identified that frequent fruit consumption has a direct positive relationship, whereas savoury snacking has a direct negative relationship, with elements of our psychological health. This study also revealed that cognitive processes may be one of the mechanisms by which our dietary intake affects our psychological health, but this appears to be limited to savoury snack foods. Given that dietary intake is associated with psychological health, a more precise understanding of how, and to what extent, our diet affects psychological health could help to inform novel nutritional approaches to enhance it. Future work should look to experimentally test causality and examine the potential options for intervention.
